# HOTAIR rs7958904 polymorphism is associated with increased cervical cancer risk in a Chinese population

**DOI:** 10.1038/s41598-017-03174-1

**Published:** 2017-06-09

**Authors:** Hua Jin, Xiaoyun Lu, Jing Ni, Jinfang Sun, Bin Gu, Bo Ding, Haixia Zhu, Chao Ma, Mengjing Cui, Yuling Xu, Zhengdong Zhang, Martin Lercher, Jian Chen, Na Gao, Shizhi Wang

**Affiliations:** 1 The Immunology Laboratory, Affiliated Tumor Hospital of Nantong University (Nantong Tumor Hospital), Nantong, China; 2 Department of Pathology, Affiliated Tumor Hospital of Nantong University (Nantong Tumor Hospital), Nantong, China; 3 Department of Gynecologic Oncology, Jiangsu Cancer Hospital, Jiangsu Institute of Cancer Research, Nanjing Medical University Affiliated Cancer Hospital, Nanjing, China; 4 Key Laboratory of Environmental Medicine Engineering, Ministry of Education, School of Public Health, Southeast University, Nanjing, China; 5 Department of Neurosurgery, Zhongda Hospital, School of Medicine, Southeast University, Nanjing, China; 6 Department of Gynecology and Obstetrics, Zhongda Hospital, School of Medicine, Southeast University, Nanjing, China; 7 Department of Environmental Genomics, Jiangsu Key Laboratory of Cancer Biomarkers, Prevention and Treatment, Cancer Center, Nanjing Medical University, Nanjing, China; 8 Department of Genetic Toxicology, the Key Laboratory of Modern Toxicology of Ministry of Education, School of Public Health, Nanjing Medical University, Nanjing, China; 9 Institute of Bioinformatics, Heinrich Heine University, Dusseldorf, Germany

## Abstract

Previously, we have identified single nucleotide polymorphisms (SNPs) rs7958904 and rs4759314 in long non-coding RNA HOX transcript antisense RNA (HOTAIR) were significantly associated with risk of colorectal and gastric cancer, respectively. Here, we aimed to investigate the association between HOTAIR SNPs and cervical cancer (CC) susceptibility. A total of 1209 cases and 1348 controls were enrolled for association study and genotyped with TaqMan allelic discrimination method. The Cancer Genome Atlas (TCGA) database was utilized for *in vivo* analysis of allele-specific HOTAIR expression. MTT assay was employed for evaluation of allele-specific cell proliferation. The rs7958904 CC genotype was related to an increased risk of cervical cancer compared with the GG/GC genotypes (OR = 1.57, 95% CI = 1.10–2.25). TCGA database showed the CC tissues with rs7958904 CC genotype had higher HOTAIR expression than those with GG genotype (*P* = 0.046). MTT assay demonstrated a growth-promoting role of rs7958904 C allele on CC cells. Further functional studies on the effect of rs7958904 on biological behavior of CC cells are needed to confirm and extend our findings. In conclusion, HOTAIR rs7958904 might influence CC susceptibility through modulation of CC cell proliferation, and could serve as a diagnostic biomarker.

## Introduction

Cervical cancer (CC) is one of the most common causes of cancer mortality for women worldwide. According to the International Agency for Research on Cancer (IARC) (GLOBOCAN 2012), an estimated of 266,000 deaths from CC occurred worldwide in 2012, accounting for 7.5% of all female cancer deaths^1^. Although infection with high risk human papillomavirus (HR-HPV) has been accepted as the major risk factor of CC, individual genetic heritability also plays a fundamental role in the development of CC^2–4^.

Long noncoding RNAs (lncRNAs) are a kind of non-coding RNAs longer than 200 nucleotides^5, 6^. The ENCODE project suggests that about 76% of the human genome is transcribed to a series of lncRNAs^7^. Recently, lncRNAs have gained more and more attention due to their complex and extensive regulatory functions^5, 6, 8^. LncRNAs functions vitally in a broad range of cellular processes, such as cell growth, survival, migration and invasion^9, 10^. Deregulated lncRNAs have been reported to be involved in pathogenesis of cancers^6, 11^. LncRNA HOX transcript antisense intergenic RNA (HOTAIR) locates in the *HOXC* locus, and involves epigenetic regulation of transcription in a 40 kb region of HOXD^10^. HOTAIR participates in the development and progression of a variety of malignances including breast cancer^10^, colorectal cancer^12^, and gastric cancer^13^. A number of studies showed that HOTAIR was overexpressed in CC and associated with progression and poor prognosis of CC and could be served as a new biomarker for overall survival^14, 15^.

Recently, considerable efforts have been made to investigate the effect of genetic variations in the lncRNA genes on the susceptibility of various tumors. Verhaegh *et al*.^16^ first reported that the tagging SNP (tagSNP) rs2839698 of H19 was significantly associated with the decreased risk of bladder cancer. Hereafter, a number of lncRNA SNPs, such as rs6434568 in the *PCGEM1* and rs11655237 in exon 4 of *LINC00673* genes, have been found to confers susceptibility to tumorigenesis^17, 18^. In our previous studies, we observed that two SNPs, rs7958904 and rs4759314, in the HOTAIR gene were significantly associated with colorectal^19^ and gastric cancer^20^, respectively. Given the important role of HOTAIR in carcinogenesis and its dysregulation in CC, we hypothesized that the HOTAIR SNPs could influence the susceptibility of CC. As a result, we identified rs7958904, located in exon 6 of HOTAIR, was significantly associated with increased risk of CC.

## Materials and Methods

### Ethics statement

The study was approved by the institutional review board of Southeast University. Each subject signed an informed consent. The research protocol was carried out in accordance with the approved guidelines.

### Study subjects

There were 1209 cases and 1348 healthy controls enrolled in this study. Among them, 571 cases and 657 controls were recruited from hospitals in Nanjing and Wuxi cities between January 2007 and December 2010, which has been described elsewhere^21^. The other 638 cases and 691 controls were recruited from Nantong hospitals between January 2009 and December 2014. The patients were all histologically confirmed and those who had metastasized cancers from other origin were excluded. The healthy controls were enrolled from people seeking for health care in the same hospitals. They were genetically unrelated to the cases and had no history of gynecologic tumors.

### SNP selection and Genotyping

The overall flow of SNP selection was described in our previous study^19^. Briefly, the three tagging SNPs (i.e., rs4759314, rs7958904, and rs874945) were picked up using Haploview 4.0 software and the threshold for analysis was set as *r* ^2^ > 0.8. The genotypes of all SNPs were determined by TaqMan allelic discrimination methods. The random 10% of samples were repeatedly genotyped and the results were 100% concordant.

### Genotype imputation

The Cancer Genome Atlas (TCGA) SNP array genotyping has been done using Affymetrix Genome-wide human SNP array 6.0^22^. Imputation for the three SNPs was performed using IMPUTE2 v2.3.2 with the 1000 Genomes Phase 3 as reference data.

### Cell proliferation assay

The assay was performed as described previously^19^ with a slight modification: approximately 5 × 10^3^ and 2 × 10^3^ transfected HeLa and SiHa cells, respectively, were plated in 96-well plates, and their proliferation rate was assessed using MTT at the time points of 0, 48, 72 h. The absorbance was measured at 552 nm using a spectrophotometer (Bio-Red-680, Bio-Red, USA).

### Statistical analyses

Hardy-Weinberg equilibrium (HWE) for each SNP among the controls was tested using a goodness-of-fit χ2 test. The Pearson χ2 test was used to test the differences in the frequency distribution of alleles and genotypes of SNPs. The association between SNPs and risk of CC was evaluated by multivariate unconditional logistic regression analyses with odds ratios (ORs) and 95% confidence intervals (95% CIs). The adjusted covariates included age, parity, and menopausal status. Data with homogenous variances were analyzed by using the t-test or ANOVA with Tukey-HSD multiple comparison test. In case of inequality of variances, data would first be subjected to log2 transformation, and then be handled with t-test or ANOVA if the new resultant variances were equal. Otherwise, the difference would be analyzed with Wilcoxon rank sum test. All analyses were performed using R 3.2.5 and Stata 11.0 (StataCorp LP, College Station, TX) and a *P* value of less than 0.05 was considered statistically significant.

## Results

### Association of HOTAIR tagging SNPs and CC susceptibility

The genotype frequencies of 3 tagging SNPs (i.e., rs4759314, rs7958904 and rs874945) among the controls were all in accordance with HWE (*P* = 0.294, 0.083 and 0.757, respectively). As shown in Table 1, the difference of genotype distribution of rs7958904 between the case and controls was of statistical significance (*P* = 0.029). The CC genotype was associated with a 1.58-fold increased risk (95% CI = 1.10–2.28; Table 1) of CC as compared with the GG genotype. The GC genotype conferred no significant risk of CC compared with the GG genotype (adjusted OR = 1.01, 95% CI = 0.85–1.21; Table 1). When a recessive model was employed, the CC genotype was associated with a 1.57-fold increased risk (95% CI = 1.10–2.25) of CC as compared with the GG/GC genotypes (Table 2). The difference of the rs4759314 genotype distribution was Quasi significant between the cases and controls (*P* = 0.071; Table 1). Further studies with large samples are warranted to re-confirm the association of rs4759314 with CC risk. However, no significant association was observed between the rs874945 polymorphism and risk of CC (*P* = 0.434).

**Table 1:** Association of HOTAIR SNPs with risk of CC.

SNPs	Genotype	Cases (n = 1209)		Controls (n = 1348)		*P*	Adjusted OR (95% CI)^a^
		n	%	n	%		
rs4759314	AA	1012	86.2	1162	89.1	0.071	1.00 (Ref)
	AG	158	13.5	140	10.7		1.28 (0.99–1.66)
	GG	4	0.3	2	0.2		1.50 (0.26–8.69)
rs7958904	GG	640	55.5	735	56.9	0.029	1.00 (Ref)
	GC	427	37.0	494	38.2		1.01 (0.85–1.21)
	CC	86	7.5	63	4.9		1.58 (1.10–2.28)
	GG/GC	1067	92.5	1229	95.1	0.008	1.00 (Ref)
	CC	86	7.5	63	4.9		1.57 (1.10–2.25)
rs874945	GG	745	63.6	852	66.1	0.434	1.00 (Ref)
	GA	383	32.7	394	30.6		1.10 (0.91–1.32)
	AA	43	3.7	43	3.3		1.26 (0.79–2.02)

^a^Adjusted for age, parity, and menopausal status in logistic regression model.

**Table 2:** Stratified analysis of rs7958904 genotypes and CC risk by selected variables.

Variables	GG/GC (cases/control)		CC (cases/control)		*P*	Adjusted OR (95% CI)^a^
	n	%	n	%		
All	1067/1229	92.5/95.1	86/63	7.5/4.9	0.008	1.57 (1.10–2.25)
Age (years)						
≤49	570/632	93.1/6.9	42/32	95.2/4.8	0.119	1.45 (0.89–2.37)
>49	497/597	91.9/95.1	44/31	8.1/4.9	0.026	1.70 (1.00–2.88)
Parity						
0–1	607/960	92.7/95.1	48/49	7.3/4.9	0.036	1.60 (1.05–2.46)
≥2	450/238	92.4/94.8	37/13	7.6/5.2	0.216	1.47 (0.76–2.82)
Abortion						
No	353/349	91.2/94.6	34/20	8.8/5.4	0.073	1.41 (0.75–2.66)
Yes	669/751	93.3/95.6	48/35	6.7/4.4	0.057	1.73 (1.07–2.80)
Menopausal status						
Premenopausal	605/648	92.9/95.3	46/32	7.1/4.7	0.067	1.51 (0.94–2.44)
Postmenopausal	458/490	92.0/95.3	40/24	8.0/4.7	0.028	1.64 (0.95–2.84)
Depth of invasion						
I	710/1229	93.5/95.1	49/63	6.5/4.9	0.129	1.30 (0.86–1.96)
II	314/1229	90.8/95.1	32/63	9.2/4.9	0.002	2.06 (1.29–3.31)
III/IV	31/1229	88.6/95.1	4/63	11.4/4.9	0.081	2.42 (0.81–7.21)

^a^Adjusted for age, parity, and menopausal status in logistic regression model.

### Stratified analyses of rs7958904 and risk of CC

Further stratified analyses by demographic and clinical variables showed that the association of rs7958904 with CC risk was more prominent among the subgroups of age > 49 years, parity ≤ 1, and having abortion (*P* = 0.026, OR = 1.70, 95% CI = 1.00–2.88; 0.036, 1.60, 1.05–2.46; and 0.028, 1.73, 1.07–2.80, respectively; Table 2). We also evaluated the relationship between rs7958904 and Stage II of CC, and found that the polymorphism conferred a 2.06-fold increased risk of CC (*P* = 0.002, OR = 2.06, 95% CI = 1.29–3.31).

### Allele-specific effect of rs7958904 on HOTAIR expression in CC tissues

**Figure 1:**
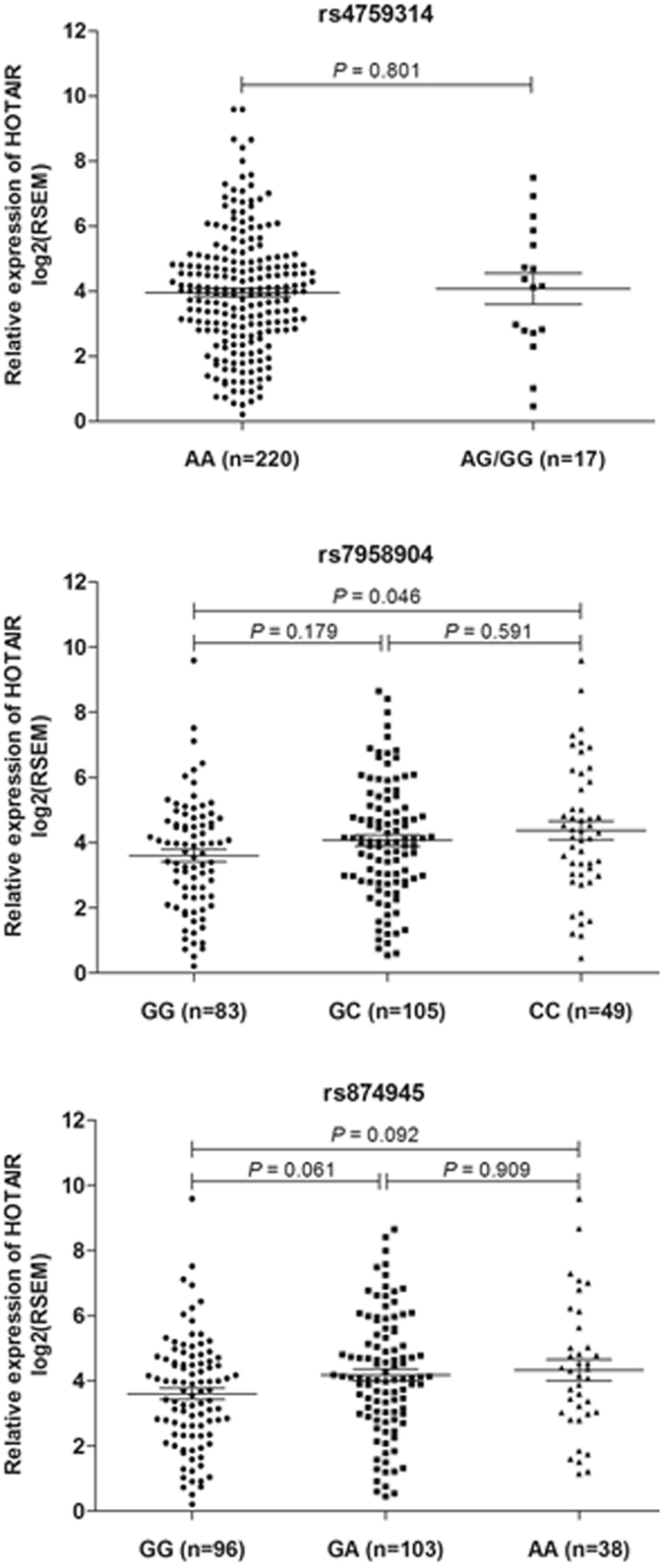
Genetic effects of SNPs on HOTAIR expression in TCGA database. Their differences were compared by ANOVA with Tukey-HSD multiple comparison test. RSEM, RNA-seq by Expectation- Maximization.

We next investigated the allele-specific effects of SNPs on HOTAIR expression by exploring the TCGA database which contains SNPs and RNA-seq information for multiple tumors including CC. Because rs4759314, rs7958904 and rs874945 were not enrolled in the TCGA SNPs data (Genome-Wide Human SNP Array 6.0), we obtained imputed genotypes of the three SNPs by imputation to the 1000 Genomes Project for the TCGA CESC SNPs data. As shown in Fig. 1, there was higher HOTAIR expression in the tumors with rs7958904 CC genotype than with wild GG genotype (*P* = 0.046). Although tumors heterozygous for rs7958904 had increased expression of HOTAIR compared with homozygous tumors, the difference was not statistically significant (*P* = 0.179). In addition, we observed no allele-specific effects of rs4759314 and rs874945 on HOTAIR expression.

### Effects of HOTAIR rs7958904 on cell proliferation

**Figure 2:**
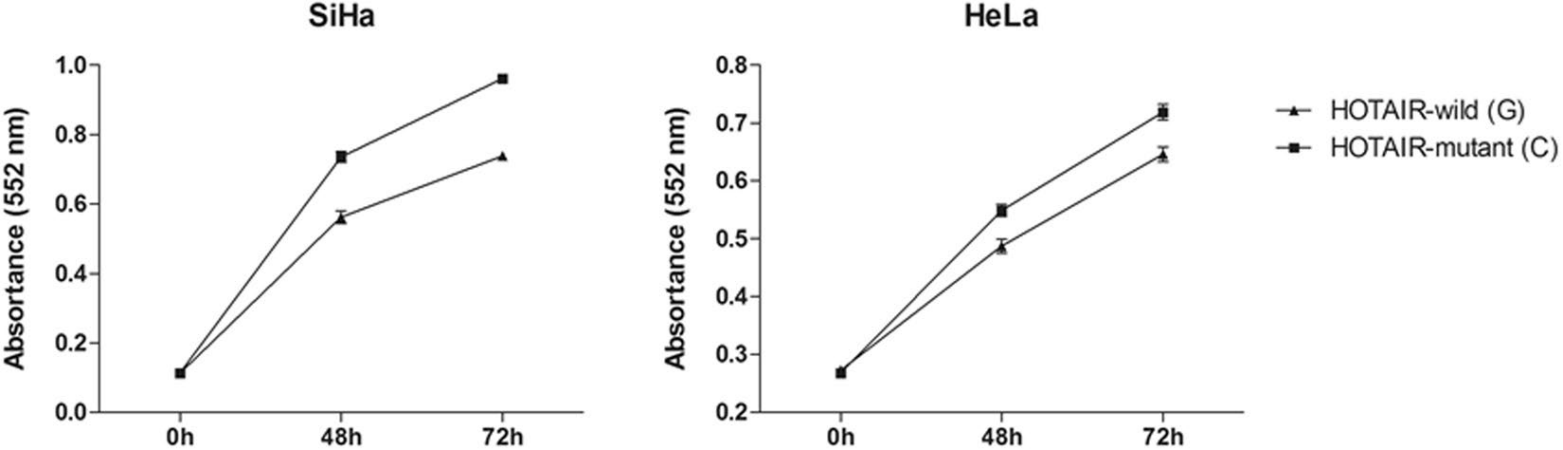
Effects of HOTAIR rs7958904 alleles on CC cell proliferation. Increased cell proliferation was observed in the CC cells transfected with plasmids containing mutant C allele over the wild G allele.

Previously, we identified a growth-inhibiting role of rs7958904 C allele in colorectal cancer LoVo cells^19^. To determine the underlying mechanism for rs7958904 conferring an increased risk of CC, we also performed a cell proliferation assay to test the effect of rs7958904 on CC cell proliferation. Interestingly, MTT assay showed higher proliferation rate of both HeLa and SiHa cells transfected with rs7958904 C allele than with G allele (Fig. 2), which was opposite to the findings in the LoVo cells.

## Discussion

In the present study, we performed a case-control study to investigate the association of 3 tagging SNPs with risk of CC, and found that the rs7958904 polymorphism conferred an increased risk of CC. Functional assays showed higher HOTAIR expression in CC tissues with rs7958904 CC genotype than with GG genotype. MTT assay demonstrated a growth-promoting role of rs7958904 C allele on CC cells.

HOTAIR has been found to be deregulated in a variety of tumors, including lung^23^, colon^12^, stomach^13^, ovarian^24^, and esophageal cancers^25^ since it was first identified in breast cancer to promote tumor invasiveness and metastasis in 2010^10^, HOTAIR is also found to be overexpressed in CC and predict a poor prognosis of patients^14, 15^. Jing *et al*.^26^ reported that HOTAIR enhanced aggressive biological behaviors and induced radio-resistance via inhibiting p21, suggesting an onco-lncRNA in CC. Accumulating evidence has suggested that genetic variants in the lncRNAs can modulate individual susceptibility to cancer^17, 27^. Zhang *et al*.^28^ first evaluated the relationship between the tagging SNPs (i.e., rs920778, rs1899663 and 4759314) of HOTAIR and risk of esophageal squamous cell carcinoma (ESCC). Thereafter, several studies have investigated the association of HOTAIR SNPs with multiple cancer susceptibility^29^.

To date, there are two studies on the association of HOTAIR SNPs and risk of CC^30, 31^. Qiu *et al*.^31^ reported that rs920778 was significantly associated with the development and progression of CC. Guo *et al*.^30^ evaluated the three tagging SNPs identified by Zhang *et al*.^28^ in relation to CC susceptibility, and also found a strong association between rs920778 and risk of CC. In the present study, we evaluated the association of three tagging SNPs (i.e., rs4759314, rs7958904 and 874945) with risk of CC, and found only rs7958904 was significantly associated with risk of CC. We previously identified rs7958904 was not markedly associated with gastric cancer^20^ but related to a decreased risk of colorectal cancer^19^. We observed a growth-promoting role of rs7958904 C allele on CC cells, which was opposed to its growth-inhibiting effect in colorectal cancer LoVo cells. The opposite effect of rs7958904 on cancer risk may reflect the complex function of HOTAIR and its genetic variation is complex and depends on cell-type context. Another explanation for rs7958904 in relation to CC susceptibility is that the real functional SNP is rs920778, which is in high LD (*r* ^2^ = 1) with rs7958904. Guo *et al*.^30^ found rs920778 T allele could enhance intronic enhancer activity and increase HOTAIR expression in CC cells. Further studies on the interaction effect of rs7958904 C allele and rs920778 T allele on biological behavior of CC cells are warranted.

The rs4759314 polymorphism locates in the first intron of HOTAIR. We previously observed a significant association between rs4759314 and risk of gastric cancer^20^. However, no significant association was observed between rs4759314 and risk of several tumors including breast, colorectal, and esophageal cancer^29^. The rs874945 polymorphism locates in the 3’ near gene of HOTAIR. In our previous studies, there was no significant association of rs874945 with risk of gastric or colorectal cancer^19, 20^. Because rs874945 and rs1899663 are in high LD (*r* ^2^ = 0.904)^20^, the association of rs1899663 with cancer susceptibility was also reviewed. No significant association was observed between rs1899663 and risk of cervical, gastric, breast, and esophageal cancer^29, 30^.

In conclusion, we identified rs7958904 in the exon of HOTAIR significantly was associated with increased risk of CC and could influence CC cell proliferation. Further functional studies on the effect of rs7958904 C allele and its interaction with rs920778 T allele on biological behavior of CC cells are are needed to confirm and extend our findings.
